# Chinese herbal medicine for chronic pain: a bibliometric analysis based on integrated databases (2011–2024)

**DOI:** 10.3389/fmed.2025.1642093

**Published:** 2025-08-08

**Authors:** Xiangyu Wang, Yongfang Li, Haojin Cheng, Hao Wu, Jiuyi Yang

**Affiliations:** ^1^Hospital of Chengdu University of Traditional Chinese Medicine, Chengdu, China; ^2^Chengdu University of Traditional Chinese Medicine, Chengdu, Sichuan China; ^3^Chongqing Traditional Chinese Medicine Hospital, Chongqing, China

**Keywords:** Chinese herbal medicine, chronic pain, visualized analysis, primary care, anti-inammatory, clinical trial

## Abstract

**Background:**

Chronic pain has become an increasingly prevalent issue in primary care. Current management in modern medicine for chronic pain often remains unsatisfactory. Chinese herbal medicine has gained growing recognition as a complementary approach. However, there is still a lack of methodical bibliometric analysis in this field. This study aims to review the research landscape, assess the current state of research, and explore prospects through a bibliometric analysis of Chinese herbal medicine for chronic pain in primary care.

**Methods:**

Relevant literature published between 2011 and 2024 was retrieved from the Science Citation Index Expanded within the Web of Science Core Collection for this primary bibliometric analysis. Additionally, clinical trials from the PubMed database were selected to evaluate clinical research progress. Visualization tools, including CiteSpace and VOSviewer, were employed to analyze journals, institutions, keywords, publication trends, keyword bursts, and reference bursts. The entire research process adhered to the BIBLIO checklist.

**Results:**

A total of 471 publications were included in the analysis, indicating a steady annual increase in research on Chinese herbal medicine for chronic pain. The *Journal of Ethnopharmacology* was identified as the most frequently cited and co-cited journal. High-output institutions were primarily located in China’s developed coastal regions. A total of 2,882 researchers contributed to these studies, with Lu Aiping identified as the most prolific author. Current research hotspots focus on chronic pain associated with arthritis and cancer. Network pharmacology and molecular docking have emerged as key methodologies. Notably, *Tripterygium wilfordii Hook.f Sophora flavescens Aiton, Conioselinum anthriscoides “Chuanxiong,” and Paeonia lactiflora Pall* were among the most studied herbs. The anti-inflammatory and analgesic mechanisms of traditional Chinese medicine represent a major research frontier. In clinical research, key focus areas include methodological refinement, real-world evidence studies, clinical trials on cancer pain, and comparative and integrative approaches combining Chinese herbal medicine with modern medical management.

**Conclusion:**

The study provides a comprehensive bibliometric overview of the current status and research hotspots in the field, offering valuable insights for future investigations. The findings highlight the growing academic interest and increasing international recognition of Chinese herbal medicine in this field within primary care settings.

## 1 Introduction

The International Association for the Study of Pain defines chronic pain as pain that persists for more than 3 months, significantly impacting individuals’ physical and mental wellbeing (1). With the increasing prevalence of chronic non-communicable diseases and the continuous aging of the global population, chronic pain has become a major public health concern. It not only diminishes quality of life but can also shorten life expectancy (2). Chronic pain impairs self-care capacity, emotional wellbeing, and cognitive function (3). Notably, nearly one-fifth of adults worldwide suffer from chronic pain (4), indicating that its effects extend beyond individuals to exert substantial economic and social burdens. For instance, in Europe, the economic cost of chronic pain has been estimated at approximately 3% of the annual Gross Domestic Product (GDP) (5). As a result, the management of chronic pain has become a pressing issue in the field of primary care.

In modern medicine, the primary pharmacological management for chronic pain includes non-steroidal anti-inflammatory drugs, acetaminophen, and opioids. However, these management approaches are far from ideal (6). From an efficacy standpoint, these medications often fail to provide adequate and sustained relief. From the perspective of safety, long-term use of anti-inflammatory drugs can lead to gastrointestinal and systemic adverse reactions (7), while prolonged acetaminophen use is associated with liver toxicity (8). Opioids, although effective in some cases, are accompanied by significant risks, including tolerance, addiction, and respiratory depression (9). Consequently, there is a growing need to explore new therapeutic strategies for chronic pain, especially in primary care settings (10).

Chinese herbal medicine (CHM), a fundamental component of traditional Chinese medicine (TCM), is derived from centuries of clinical practice and empirical knowledge. It has demonstrated unique advantages in managing chronic pain. On one hand, the therapeutic efficacy of CHM has been substantiated in various pain conditions, including inflammatory, cancer-related, and neuropathic pain (11–14). On the other hand, when used in combination with modern analgesics, CHM can reduce the required dosage of conventional drugs, thereby minimizing the risk of related side effects (15). Recent studies also suggest that patients in primary care increasingly prefer complementary and alternative medicine approaches—particularly TCM—for managing chronic pain (16).

Although research on CHM for chronic pain is well-established, a comprehensive review of research trends, development status, and emerging hotspots is still lacking. While bibliometric studies have been conducted on acupuncture (17), moxibustion (18), and tuina (19) for chronic pain, similar analyses focusing specifically on CHM are rare. Therefore, a bibliometric analysis is urgently needed to evaluate the research landscape, identify current trends, and anticipate future directions in the application of CHM for chronic pain. This will help fill the existing knowledge gap and provide valuable insights for scholars and practitioners, ultimately advancing research and clinical practice in this important area of primary care.

## 2 Methods

### 2.1 Data sources and search strategy

The Science Citation Index Expanded of the Web of Science Core Collection was searched for this study. As a highly authoritative digital literature database, Web of Science is widely recognized by researchers and is considered the most appropriate database for foundational bibliometric analysis.

To ensure comprehensive coverage of relevant literature in this field, a carefully designed search strategy was employed. A total of 1,310 English-language publications were retrieved from January 2011 to July 2024. These 14 years were selected to capture recent developments and high-impact publications, enabling robust trend analysis and the identification of emerging research frontiers. The specific search terms and strategy are provided in Supplementary Table 1.

To enhance the scientific rigor of this study and avoid the limitations of relying on a single database, PubMed was also selected as a complementary data source. Recognized as a high-quality database in the biomedical field, PubMed was used to capture literature on clinical research progress related to CHM for chronic pain. This additional data source allows for a more objective and comprehensive understanding of research trends and hotspots in clinical studies (clinical trials). The detailed PubMed search strategy is provided in Supplementary Table 2.

### 2.2 Literature screening inclusion and exclusion criteria

A methodical literature screening process was conducted to identify studies eligible for inclusion in this review. The screening process adhered to the BIBLIO checklist (Supplementary Table 3) for reporting bibliometric reviews of biomedical literature, ensuring methodological transparency and rigor.

The screening was performed in two phases. In the first phase, two independent reviewers (the first author, Xiangyu Wang, and the second author, Yongfang Li) screened the titles and abstracts of all retrieved records based on predefined inclusion and exclusion criteria. In the second phase, studies deemed potentially eligible underwent full-text review by the same reviewers. Any discrepancies or disagreements were resolved through discussion or, if necessary, consultation with a third reviewer (the corresponding author, Jiuyi Yang).

After completing the literature selection from both databases, EndNote (version 21.2) was employed as a reference management tool to identify and remove duplicates among the studies ultimately included from the Web of Science and PubMed databases. Subsequently, all records were manually checked for accuracy. Most duplicate entries were found among review articles. However, since only clinical trials were included from the PubMed database, this did not affect the final literature selection. In other words, there was no overlap between the final sets of included articles from the two databases.

**FIGURE 1:**
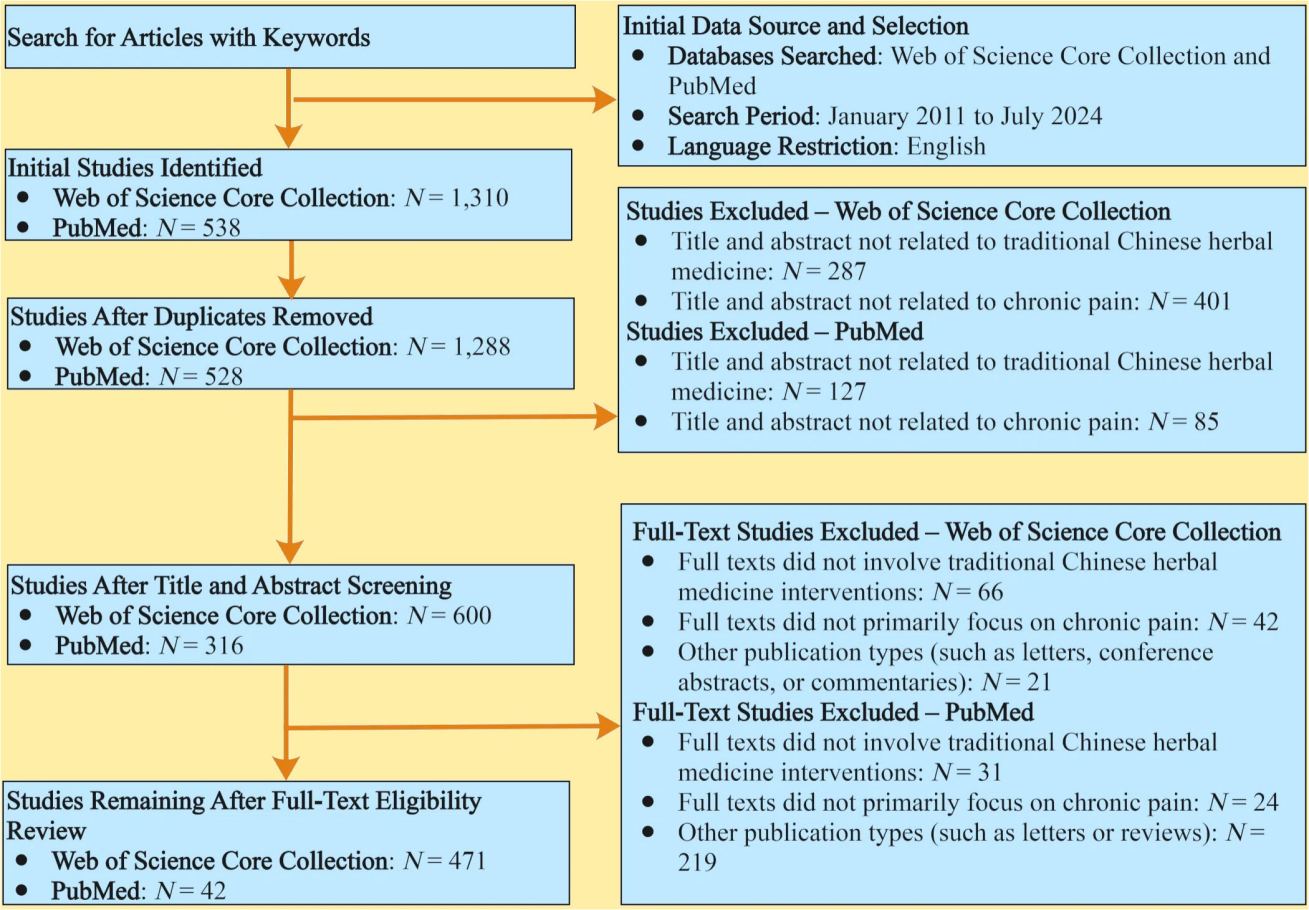
The process of filtering literature for methodical bibliometric mapping and review. Studies were included if they met the following criteria: (1) focused on chronic pain-related diseases or symptoms as the primary condition under investigation; (2) evaluated Chinese herbal medicine interventions as the primary management approach; (3) Were published as original research articles or literature reviews (Web of Science Core Collection database) or as clinical trials (PubMed database). Studies were excluded if they: (1) did not primarily focus on chronic pain conditions; (2) did not involve CHM interventions; (3) where categorized under other publication types, such as letters, conference abstracts, or commentaries (Web of Science Core Collection database), or as letters or reviews (PubMed database).

The detailed workflow of the screening process is illustrated in Figure 1.

### 2.3 Data analysis

Bibliometric analysis is a quantitative method for reviewing and evaluating existing literature in a given field. It was first introduced in 1969 (20) and has since become widely used for trend analysis and knowledge mapping (21). By extracting key bibliometric indicators—such as annual publication volume, journals, institutions, authors, keywords, and cited references—researchers can gain insight into the development trajectory, current research focus, and prospects of a field. When combined with modern visualization tools, bibliometric results can be presented in a more intuitive and informative manner.

In this study, visualizations were generated using CiteSpace (version 5.7.R5) and VOSviewer (version 1.6.20), both of which offer complementary capabilities.

CiteSpace employs set-theoretical algorithms to measure the similarity of knowledge units. Its time-slicing function is especially useful for tracking the evolution of research themes over time (22). CiteSpace was used to visualize publication output trends, keyword bursts, and reference bursts. Parameters were configured as follows: time slicing from 2011 to 2024, 1 year per slice, term sources (all selected), node types (one at a time), pruning with the default Cosine similarity threshold.

VOSviewer, on the other hand, applies probabilistic algorithms to generate co-occurrence and co-citation networks. It offers three main visualization modes: Network Visualization, Overlay Visualization, and Density Visualization, each of which is designed to be clear and aesthetically appealing (23). VOSviewer was used in this study to map journals, institutions, and keywords. These visualizations typically include three core elements: nodes, links, and colors. In keyword co-citation analysis, for example, the size of a node indicates co-citation frequency, the thickness of a link indicates the strength of co-occurrence between keywords, and color distinguishes thematic clusters.

### 2.4 Ethical considerations

This study is based solely on the analysis of publicly available literature and metadata and does not involve human subjects, interventions, or the collection of private or sensitive information. Therefore, ethical approval was not required from an institutional review board. The research adheres to the ethical principles of privacy, confidentiality, and non-maleficence.

## 3 Results

Given the distinct strengths of the two databases employed in this study, we adopted a dual-source strategy. The Web of Science Core Collection—renowned for its broad, multidisciplinary coverage—was used as the primary data set for baseline bibliometric analyses. To complement these findings, the PubMed database—widely regarded as a high-quality repository of biomedical literature—was queried specifically for clinical research progress, with an emphasis on clinical trials that illuminate current hotspots and emerging frontiers in the field.

By integrating and cross-validating data from these complementary sources, the study achieves both breadth and depth, thereby strengthening its scientific rigor. For clarity: (1) Analysis of Clinical Trial Progress (data derived from PubMed); (2) All other bibliometric analyses (data sourced from the Web of Science Core Collection).

### 3.1 Publication output trend

A total of 471 articles were included in this study, authored by 2,882 researchers affiliated with 591 institutions across 31 countries. These publications appeared in 123 journals and collectively cited 22,718 references from 5,433 different journals.

**FIGURE 2:**
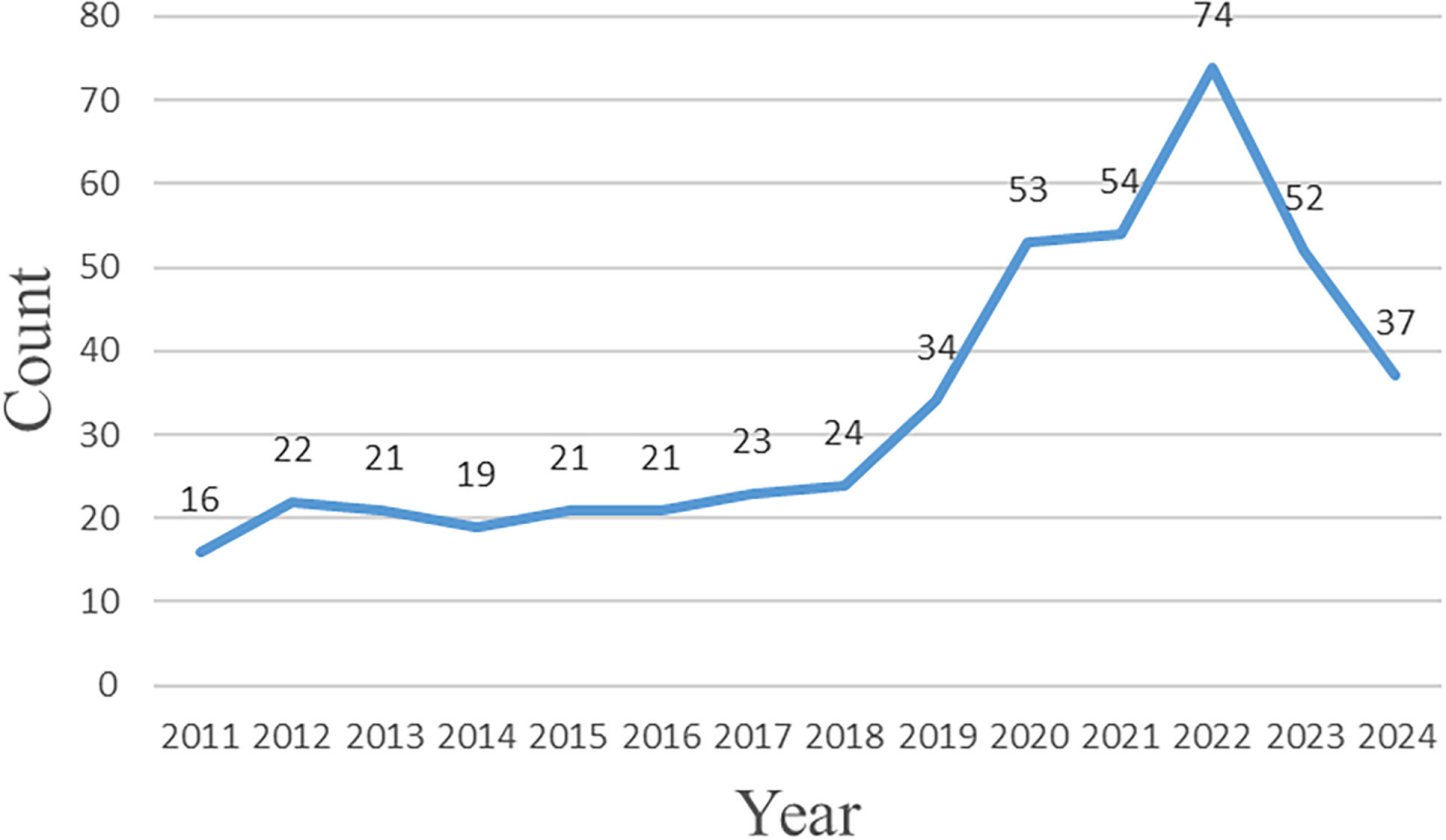
Trend in annual publication volume of academic literature.

As illustrated in Figure 2, research on CHM for chronic pain has experienced rapid growth since 2018, reaching a peak in 2022 with 74 publications. The upward trend continued into 2023, with the number of publications exceeding 50. As of July 2024, 37 articles have already been published, indicating that the total output for the year is likely to remain high.

These findings demonstrate that CHM in the context of chronic pain has attracted increasing scholarly attention in recent years, emerging as a significant research hotspot within the broader field of chronic pain management.

### 3.2 Analysis of journals and cited journals

#### 3.2.1 Analysis of journals

Understanding the core journals in a specific field helps researchers align their work with publication trends, thereby identifying prevailing research hotspots and directions. Moreover, knowledge of journal rankings provides valuable guidance for manuscript submission.

According to Bradford’s Law, the distribution of journal publication in a given field follows a 1:n:n^2^ ratio among core, related, and peripheral journals. Applying this model, core journals were defined as those publishing more than 30 articles. Based on this threshold, there are 4 core journals, 20 related journals, and 99 peripheral journals in this field. Table 1 presents the identified core journals.

**TABLE 1:** The core journals.

Journals	Documents	Citations	Average citations	Journal citation reports quartile	Impact factor
*Journal of Ethnopharmacology*	68	1,588	23.35	Q1	4.80
*Frontiers In Pharmacology*	49	998	20.37	Q1	4.40
*Evidence-Based Complementary And Alternative Medicine*	40	682	17.05	Q3	2.65
*Medicine*	33	104	3.15	Q2	1.40

As shown in Table 1, the *Journal of Ethnopharmacology* ranks first in terms of the number of publications, total citations, and average citations. It is classified in the Q1 quartile, reflecting its high impact in the field. The second-ranking journal, *Frontiers in Pharmacology*, also falls within the Q1 quartile. Both journals emphasize pharmacological research, highlighting that investigations into the mechanisms of CHM in chronic pain management occupy a central role in this research domain.

#### 3.2.2 Analysis of co-cited journals

**FIGURE 3:**
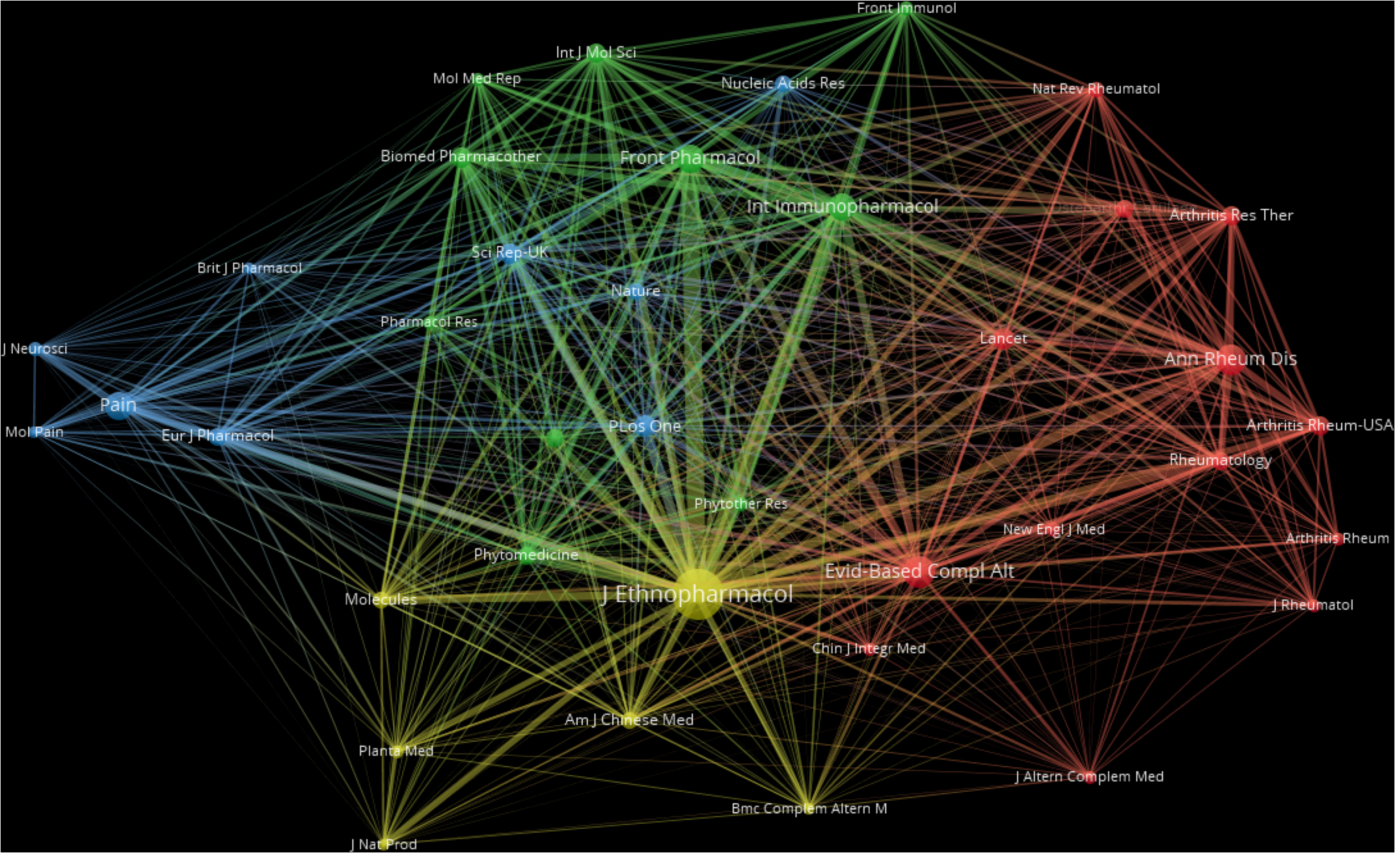
Network visualization of co-cited journals. Using VOSviewer, a co-citation map was generated for 38 journals with over 100 co-citations. The size of each node corresponds to the journal’s co-citation frequency, with larger nodes indicating higher volumes of co-citations. The thickness of the connecting lines reflects the strength of association between journals, where thicker lines represent a greater number of co-citations. The colors of the nodes and lines indicate distinct clusters, highlighting thematic relationships and groupings among the journals.

Co-citation analysis of journals is a powerful bibliometric method for identifying interconnected academic disciplines, visualizing scholarly communication networks, and informing comprehensive literature reviews. Table 2 lists the top 10 co-cited journals, and Figure 3 provides a visual representation of co-citation relationships.

**TABLE 2:** The top 10 co-cited journals.

Journals	Co-citations	Journal citation reports quartile	Impact factor
*Journal of Ethnopharmacology*	781	Q1	4.80
*Evidence-Based Complementary and Alternative Medicine*	408	Q3	2.65
*Annals of the Rheumatic Diseases*	388	Q1	20.30
*Pain*	351	Q1	5.90
*Frontiers in Pharmacology*	334	Q1	4.40
*International Immunopharmacology*	318	Q1	4.80
*PLOS ONE*	240	Q1	2.90
*Lancet*	229	Q1	98.40
*Scientific Reports*	222	Q1	3.80
*Biomedicine & Pharmacotherapy*	213	Q1	6.90

Among the most frequently co-cited journals, the *Journal of Ethnopharmacology* again ranks highest, underscoring the foundational role of pharmacological mechanism studies in this area. The second most co-cited journal is *Evidence-Based Complementary and Alternative Medicine*, reflecting the growing scholarly interest in CHM as a complementary therapy for chronic pain. The third-ranking journal, *Annals of the Rheumatic Diseases*, suggests that the application of CHM in the management of rheumatic disease-related chronic pain constitutes an important subfield and research hotspot.

### 3.3 Analysis of institutions

To identify the most productive institutions in this field and explore their collaborative networks, this study lists the top 10 institutions by publication volume (Table 3).

**TABLE 3:** The top 10 institutions in the number of publications.

Institutions	Documents	Citations	Average citation
China Academy of Chinese Medical Sciences	42	716	17.05
Guangzhou University of Chinese Medicine	34	219	6.44
Shanghai University of Traditional Chinese Medicine	32	437	13.66
Beijing University of Chinese Medicine	31	332	10.71
Anhui University of Chinese Medicine	19	151	7.95
Hong Kong Baptist University	17	290	17.06
Zhejiang Chinese Medical University	16	153	9.56
Chengdu University of Traditional Chinese Medicine	16	220	13.75
China Medical University	15	308	20.53
Fujian University of Traditional Chinese Medicine	15	179	11.93

The institution with the highest number of publications and citations is the China Academy of Chinese Medical Sciences, with 42 papers, accounting for 8.9% of the total publications. China Medical University holds the highest average citation count, at 20.53. Among the top 10 institutions by publication volume, all except Anhui University of Chinese Medicine and Chengdu University of Traditional Chinese Medicine are located in China’s economically developed coastal regions. However, according to a 2022 survey (24), the prevalence of chronic pain is significantly higher in central and western provinces such as Sichuan, Chongqing, and Guizhou. Additionally, regional variations in TCM management exist. This highlights a clear regional imbalance in research on CHM for chronic pain management.

This also indirectly suggests that the need for primary care using CHM to manage chronic pain in central and western China has not been fully recognized or adequately addressed. Therefore, further research is urgently needed in these underserved regions.

**FIGURE 4:**
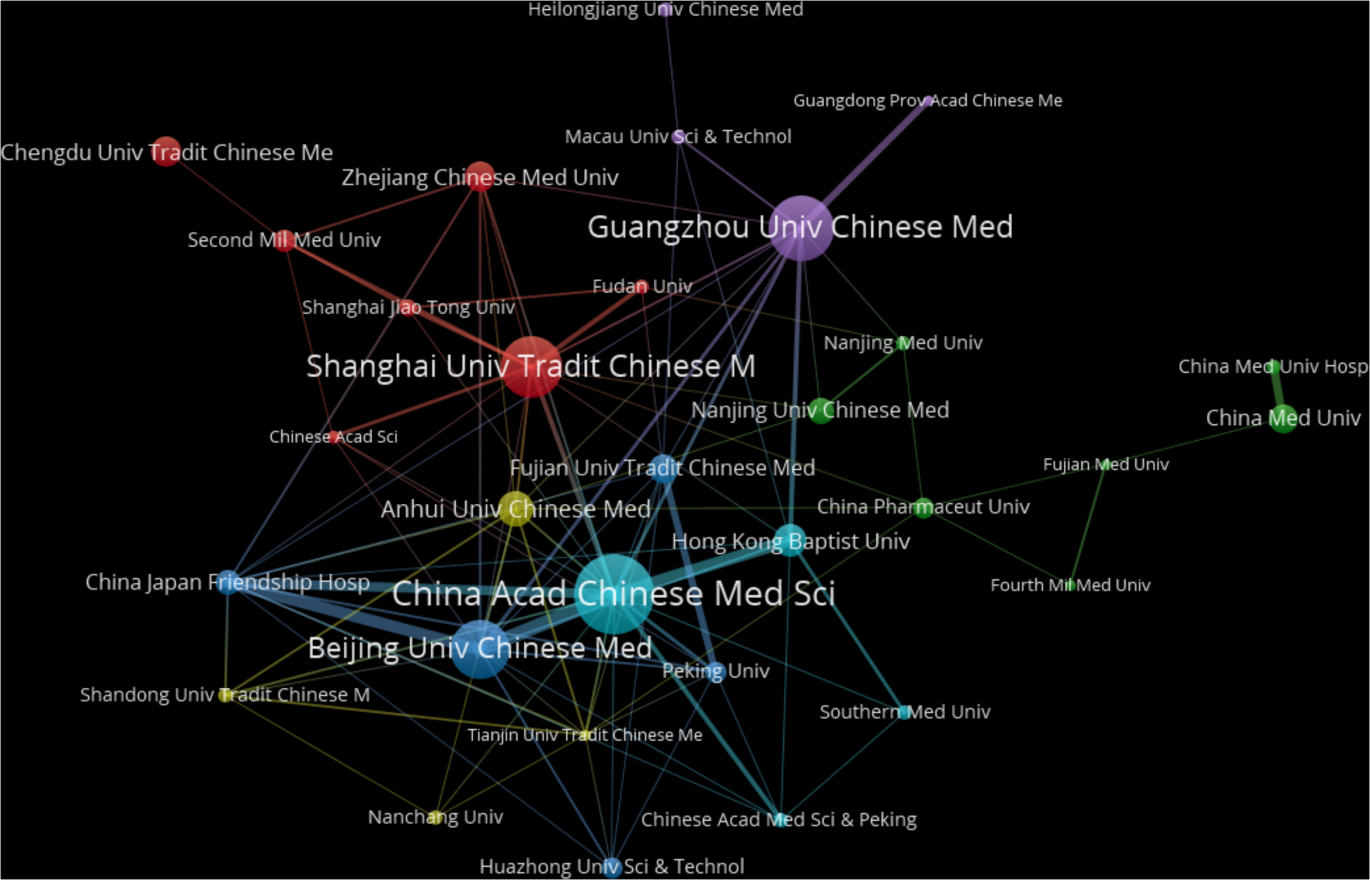
Network visualization of institutions. In this figure was created to further understand and provide a more intuitive visual analysis of the collaborative relationships between high-productivity institutions. VOSviewer was used to create a visual map of the collaboration network among 32 institutions with more than 5 publications. The size of the nodes depends on the publication frequency of the institutions; the larger the circle node, the higher the publication volume. The thickness of the connecting lines represents the strength of the association. Thicker lines indicate a higher number of collaborative publications between the connected institutions. The colors of the nodes and lines represent their respective clusters.

As illustrated in Figure 4, the distribution of publishing institutions is highly imbalanced, exhibiting a pronounced top-tier effect. Most publications originate from institutions in coastal areas, and these institutions demonstrate stronger inter-institutional collaboration. In contrast, institutions in central and western China not only have lower publication volumes but also occupy relatively marginal positions in the collaborative network.

### 3.4 Analysis of authors

Identifying the most influential scholars in a research field helps to uncover prevailing themes and emerging research directions. Accordingly, this study highlights the top five authors based on publication volume, as shown in Table 4.

**TABLE 4:** The top five authors in the number of publications.

Author	Documents	Citations	Average citation
Lu Aiping	16	295	18.44
Liu Jian	13	102	7.85
Lu Cheng	10	198	19.80
Lin Na	8	170	21.25
Jiang Miao	6	180	30.00

Among these scholars, Lu Aiping ranks first in both total number of publications (16) and total citation count (295). Jiang Miao leads in terms of average citations per publication, with an average of 30 citations. Notably, both researchers primarily focus on the use of CHM for managing chronic pain associated with rheumatic diseases, suggesting that this subfield represents a major area of scholarly interest and research activity.

### 3.5 Analysis of keywords

#### 3.5.1 Keyword selection and ranking

Bibliometric keyword analysis is essential for mapping the developmental trajectory of a research field, identifying current research hotspots, and forecasting future trends. High-frequency keywords serve as a critical foundation for this process, offering insight into the core themes of the literature.

According to Price’s Law (25), the minimum frequency (*m*) for a keyword to be considered high-frequency is calculated as:


$$m=0.749\times \sqrt{{\text{n}}_{\text{max}}}$$

**FIGURE 5:**
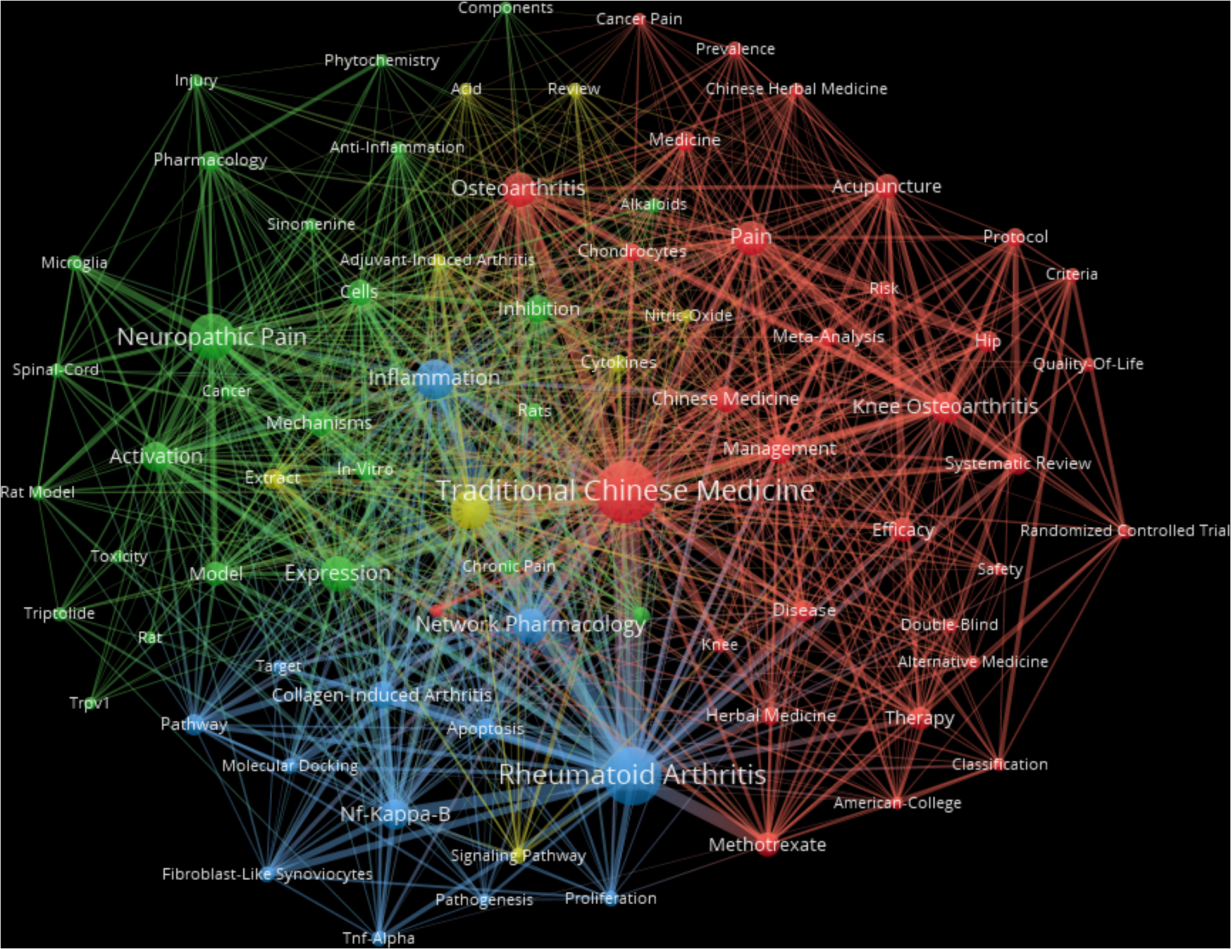
Network visualization for keywords. To present the network of relationships between keywords, identify research hotspots, and clarify research categories more comprehensively and directly, in this figure was created as a visualization map. The visualization tool VOSviewer was used to create a co-occurrence map of keywords with a frequency of 9 and above. The size of the nodes depends on the frequency of the keyword occurrence; the larger the circle node, the higher the frequency of the keyword occurrence. The thickness of the connecting lines represents the strength of the association. Thicker lines indicate a higher number of co-occurrences of the connected keywords within the same article. The colors of the nodes and lines represent their respective clusters.

Where n_max_ represents the occurrence of the most frequent keyword. In this study, the highest-frequency keyword appeared 128 times, yielding a minimum threshold of approximately 8.47. To ensure strong relevance, the minimum frequency for inclusion as a high-frequency keyword was set at 9. Based on this criterion, a keyword co-occurrence map was generated (Figure 5).

The top 10 high-frequency keywords are presented in Table 5. The four most frequently occurring keywords are: rheumatoid arthritis (172), traditional Chinese medicine (128), neuropathic pain (75), and inflammation (59).

**TABLE 5:** The top 10 keywords in terms of frequency.

Keyword	Occurrences	Total link strength
Rheumatoid arthritis	172	669
Traditional Chinese Medicine	128	485
Neuropathic pain	75	215
Inflammation	59	247
Expression	50	200
Network pharmacology	48	210
Osteoarthritis	47	187
Pain	47	187
Knee osteoarthritis	40	176
Nuclear Factor Kappa-B	39	172

These findings indicate that current research hotspots primarily center on the application of CHM for managing inflammatory conditions, rheumatoid arthritis, and neuropathic chronic pain.

#### 3.5.2 Keyword co-occurrence analysis

As illustrated in Figure 5, the keyword co-occurrence network reveals tight interconnections between various clusters. This indicates a high degree of thematic integration across different research topics related to CHM for chronic pain, reflecting a cohesive and interrelated body of work.

#### 3.5.3 Keyword burst analysis

**FIGURE 6:**
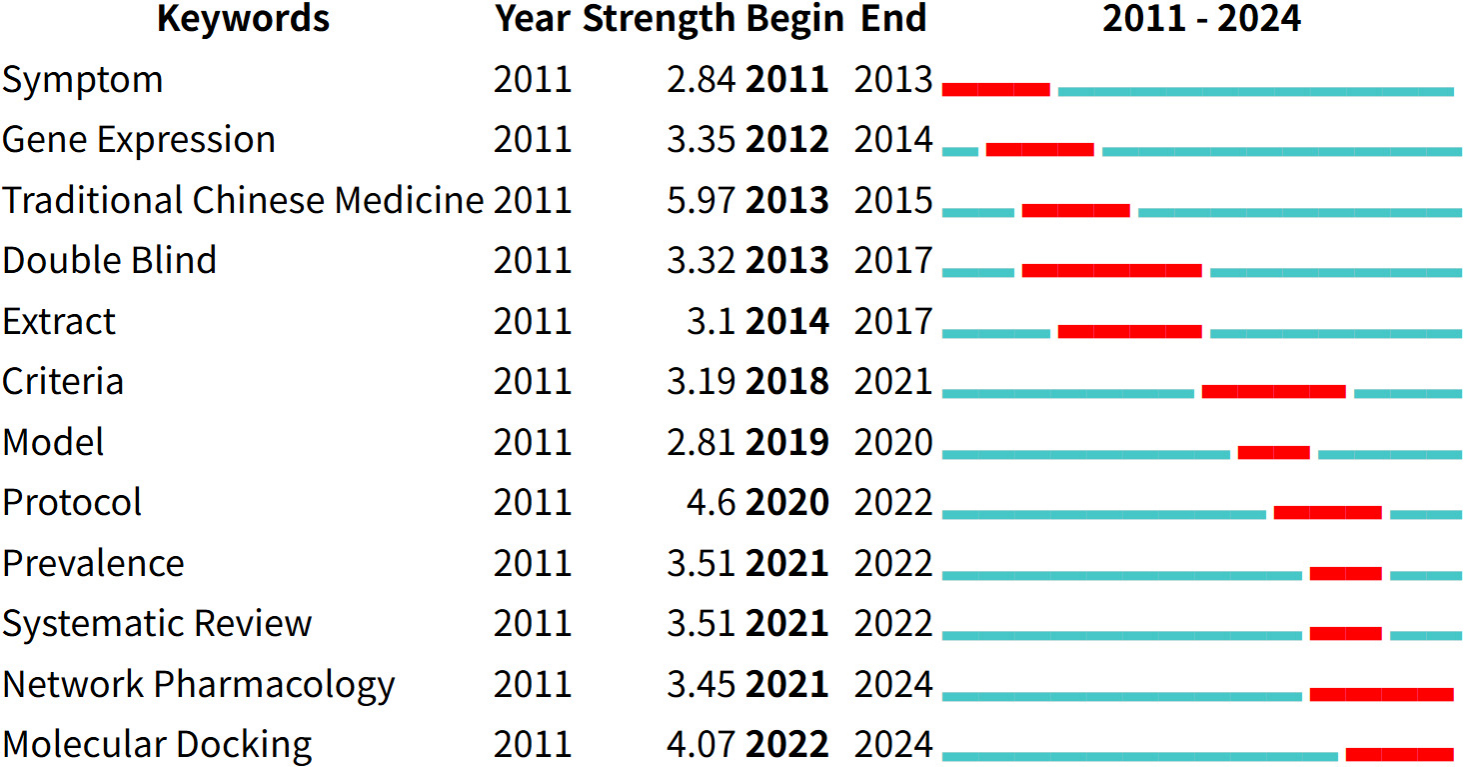
The top 12 keywords with the strongest citation bursts. CiteSpace was used to visualize the keyword bursts with the time slice set to 1 year, detecting a total of 12 keyword bursts.

Keyword burst analysis helps identify shifts in research focus over time and highlights emerging areas of interest. As shown in Figure 6, the term “traditional Chinese medicine” exhibits the strongest burst intensity (5.97), which aligns with its central role in the field.

Recent burst keywords include “network pharmacology” and “molecular docking,” both of which are expected to remain influential. These terms reflect the growing trend of applying modern technological tools to the study of CHM for chronic pain, such as for new drug development, efficacy evaluation, and mechanistic exploration. These technologies are likely to become key research frontiers shortly.

An analysis of keyword bursts also reveals a phased evolution in the field: (1) Early stage (2011–2017): Focused on theoretical groundwork, with keywords such as “gene expression” and “extract” indicating foundational research and initial exploration; (2) Mid-stage (2018–2020): Marked by standardization efforts, characterized by keywords like “criteria,” “model,” and “protocol,” reflecting the development of methodological norms; (3) Recent stage (2021–2024): Represents a phase of review and methodological innovation, featuring both retrospective analysis (“systematic review”) and the adoption of advanced tools (“network pharmacology,” “molecular docking”). This progression suggests a maturing field that is evolving from basic conceptual studies to the application of sophisticated computational and pharmacological methodologies.

### 3.6 The top 10 cited documents analysis

Analyzing citation patterns helps researchers quickly identify highly influential core papers in a given field, thereby guiding future investigations. Accordingly, this study highlights the top 10 most cited publications in the domain (Table 6).

**TABLE 6:** The top 10 documents in terms of citations.

Documents	First author	Publication year	Citations
*Triptolide: Progress on research in pharmacodynamics and toxicology*	Li Xiao Jiaoyang	2014	289
*Anti-inflammatory and immunomodulatory effects of Paeonia lactiflora Pall, a traditional Chinese herbal medicine*	He Dong-Yi	2011	260
*Medicinal plants of the genus Gelsemium (Gelsemiaceae, Gentianales)-A review of their phytochemistry, pharmacology, toxicology and traditional use*	Jin GuiLin	2014	148
*Sinomenium acutum: A review of chemistry, pharmacology, pharmacokinetics, and clinical use*	Zhao Xiangxiang	2012	125
*Mechanisms involved in the therapeutic effects of Paeonia lactiflora Pallas in rheumatoid arthritis*	Zhang Wei	2012	113
*The treatment of rheumatoid arthritis using Chinese medicinal plants: From pharmacology to potential molecular mechanisms*	Lu Shaowa	2015	111
*A Natural Isoquinoline Alkaloid With Antitumor Activity: Studies of the Biological Activities of Berberine*	Liu Da	2019	104
*A Novel Analgesic Isolated from a Traditional Chinese Medicine*	Zhang Yan	2014	80
*Chinese herbal prescriptions for osteoarthritis in Taiwan: analysis of national health insurance dataset*	Chen Fangpey	2014	71
*Topical herbal therapies for treating osteoarthritis*	Cameron Melainie	2013	70

The most frequently cited article is “*Triptolide: Progress on Research in Pharmacodynamics and Toxicology*” (26). This paper primarily explores the therapeutic mechanisms of *Tripterygium wilfordii Hook.f* in the management of some pain-related diseases, particularly in the treatment of cancer.

The second most cited article, “*Anti-inflammatory and Immunomodulatory Effects of Paeonia lactiflora Pall, a Traditional Chinese Herbal Medicine*” (27), discusses the specific anti-inflammatory and immunomodulatory mechanisms through which *Paeonia lactiflora Pall* exerts its analgesic effects.

The third most cited publication is “*Medicinal Plants of the Genus Gelsemium (Gelsemiaceae, Gentianales): A Review of Their Phytochemistry, Pharmacology, Toxicology, and Traditional Use*” (28). This review primarily summarizes the pharmacodynamic evidence of *Gelsemium elegans (Gardn. & Champ.) Benth*, a traditional analgesic herb.

The fourth most cited publication is “Sinomenium acutum: a review of chemistry, pharmacology, pharmacokinetics, and clinical use” (29). This article reviews the chemical constituents, pharmacological mechanisms, and clinical applications of *Sinomenium acutum (Thunb.) Rehd. Et Wils*, a traditional CHM commonly used in the treatment of pain associated with rheumatoid arthritis.

The fifth most cited publication is “*Mechanisms involved in the therapeutic effects of Paeonia lactiflora Pallas in rheumatoid arthritis*” (30). This article focuses on the pharmacological mechanisms by which paeoniflorin glycosides alleviate symptoms, particularly pain in patients with rheumatoid arthritis.

The sixth most cited publication is “*The treatment of rheumatoid arthritis using Chinese medicinal plants: from pharmacology to potential molecular mechanisms*” (31). As rheumatoid arthritis is characterized by chronic pain, this article discusses major CHM formulas, extracts, and compounds that are currently under investigation for their therapeutic effects against rheumatoid arthritis.

The seventh most cited publication is “*A Natural Isoquinoline Alkaloid With Antitumor Activity: studies of the Biological Activities of Berberine*” (32). This article discusses the biological activity and epigenetic effects of berberine, a primary component of *Rhizoma Coptidis*, which exhibits analgesic, anti-inflammatory, and antitumor properties.

The eighth most cited publication is “*A Novel Analgesic Isolated from a Traditional Chinese Medicine*” (33). This study applies modern characterization techniques to identify dehydrocorybulbine, a novel analgesic compound isolated from TCM, and examines its effectiveness in managing pain, particularly chronic pain.

The ninth most cited publication is “*Chinese herbal prescriptions for osteoarthritis in Taiwan: analysis of national health*” (34). This study presents a large-scale pharmacoepidemiological analysis of CHM use among osteoarthritis patients in Taiwan in 2002, with a focus on CHM’s role in chronic pain management.

The tenth most cited publication is “*Topical herbal therapies for treating osteoarthritis*” (35). This systematic review evaluates the efficacy of topical CHMs for osteoarthritis, with particular attention to their impact on managing chronic pain associated with rheumatoid arthritis.

Of the top 10 most cited papers, nine were published between 2011 and 2015. The most recent among them is “*A Natural Isoquinoline Alkaloid with Antitumor Activity: Studies of the Biological Activities of Berberine*.” The high citation count of this recent publication suggests that research on berberine has emerged as a new focal point in the field, warranting further in-depth exploration.

### 3.7 Reference bursts analysis

Reference bursts can reflect changes in the attention received by references in a particular field, thereby indicating shifts in research hotspots.

**FIGURE 7:**
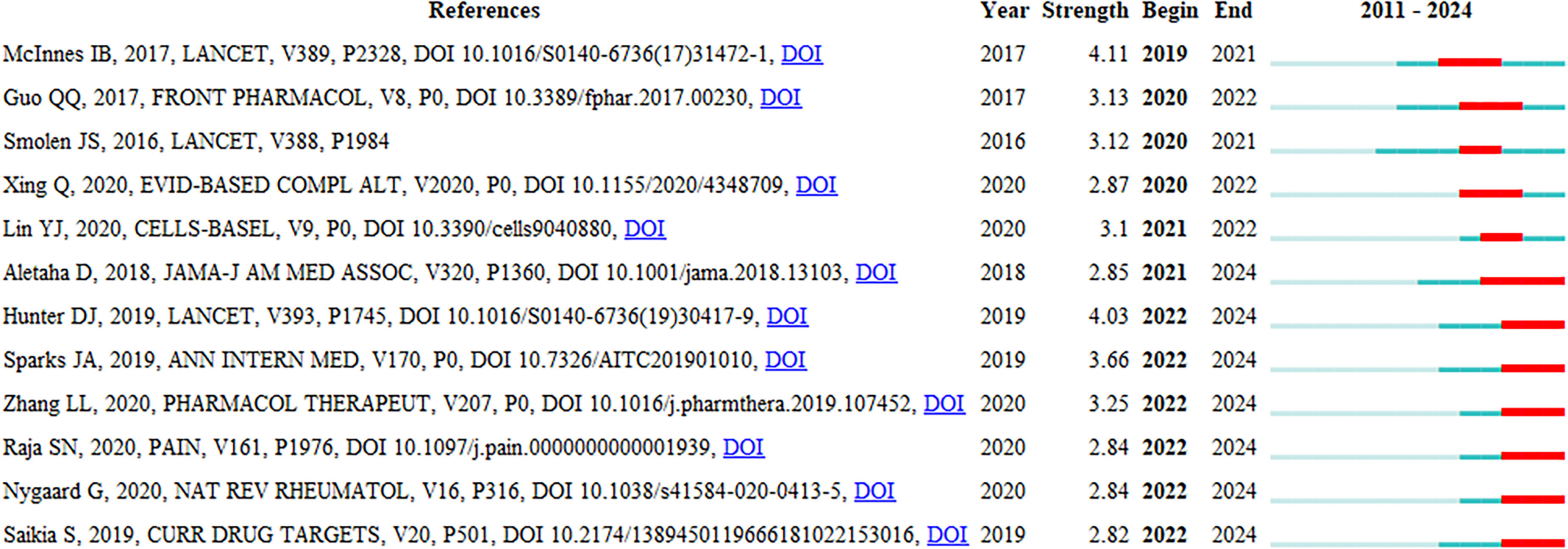
The top 12 references with the strongest citation bursts. CiteSpace was used to visualize the reference bursts with the time slice set to 1 year, detecting a total of 12 reference bursts.

As shown in Figure 7, the reference bursts from 2018 to 2024 include articles by Aletaha (36), Hunter (37), Sparks (38), Zhang (39), Raja (40), Nygaard (41), and Saikia (42). These reference bursts suggest that chronic pain management in arthritis—especially osteoarthritis and rheumatoid arthritis—has become a major focus of recent and prospective research.

### 3.8 Analysis of key CHM

Based on a combination of highly cited literature and a comprehensive review of 471 selected publications, this study identifies several key herbal medicines that represent current research hotspots and emerging frontiers in the management of chronic pain. Notably,*Tripterygium wilfordii Hook.f*, *Sophora flavescens Aiton*, *Conioselinum anthriscoides ‘Chuanxiong’*, and *Paeonia lactiflora Pall* have garnered significant scholarly attention.

The clinical application of these herbs aligns with TCM practices and is supported by findings from modern pharmacological research. These insights not only guide future scientific research in related fields but also offer valuable references for clinical application in the management of chronic pain.

### 3.9 Analysis of clinical trial progress

PubMed is recognized as a high-quality medical database that includes numerous well-conducted clinical trials. Accordingly, this study utilized PubMed to analyze the clinical progress of CHM in chronic pain management. The aim is to provide insight into current research hotspots and emerging trends for clinical researchers in this field.

**FIGURE 8:**
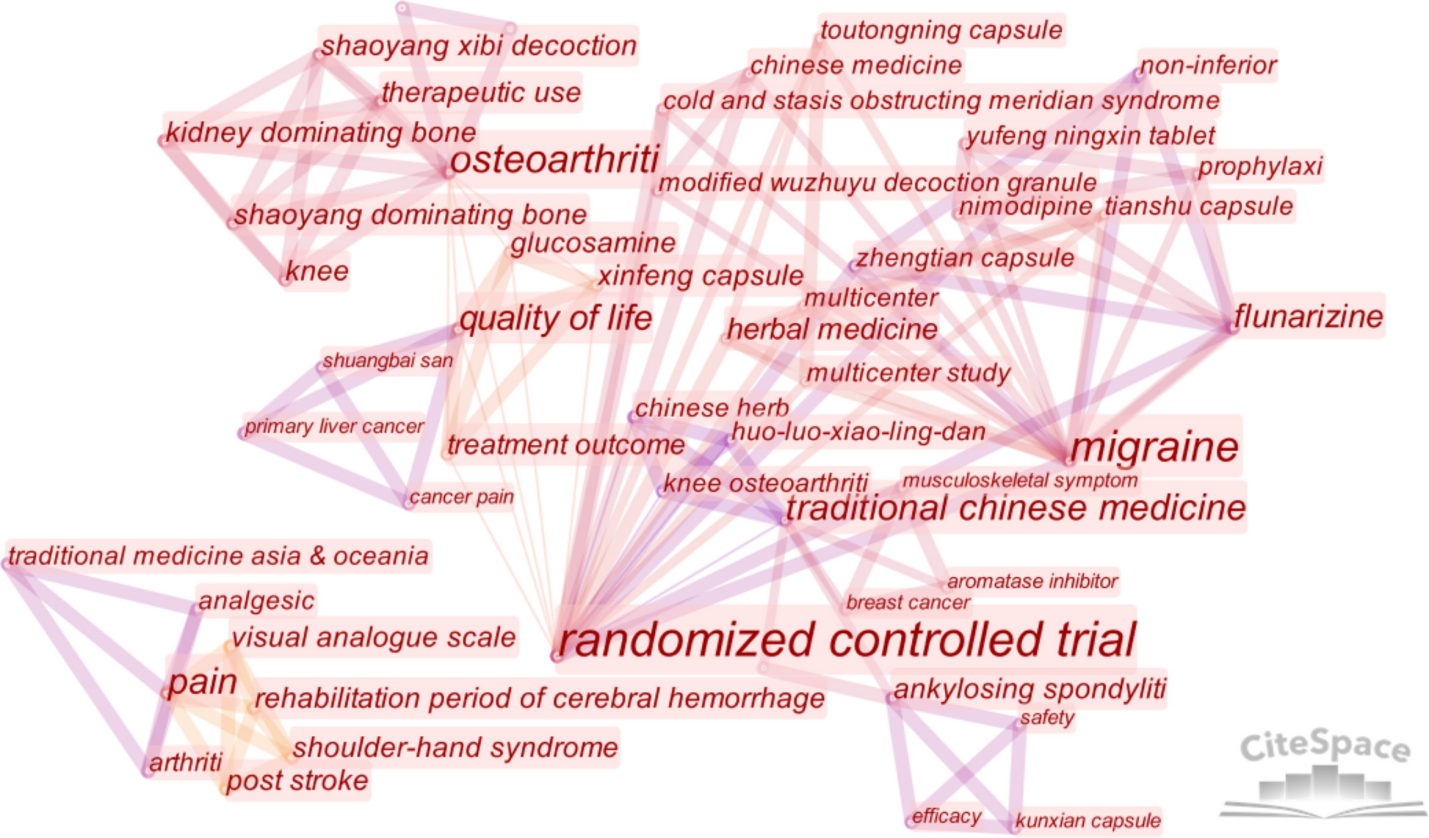
Keyword co-occurrence map of clinical trial progress.

Keywords serve as an important window for understanding the focus and frontier of a research area. By constructing a keyword co-occurrence map (Figure 8) and analyzing high-frequency terms (Table 7), this study draws several conclusions regarding the progress of clinical research on CHM in chronic pain management.

**TABLE 7:** High-frequency clinical trial keywords.

Keyword	Frequency	Degree	Centrality
Randomized controlled trial	6	21	0.91
Traditional Chinese Medicine	3	9	0.43
Osteoarthritis	3	12	0.38
Quality of life	2	8	0.18
Ankylosing spondylitis	2	5	0.18
Migraine	5	15	0.14
Flunarizine	2	7	0.07
Pain	2	9	0.03
Herbal medicine	2	6	0

#### 3.9.1 Advancing toward high-quality clinical trial design

The term “randomized controlled trial” (RCT) ranks highest in both frequency and centrality, appearing at the core of the co-occurrence map and showing close association with multiple topics. This indicates a growing emphasis in the field on high-quality, evidence-based clinical trial designs, exemplified by RCTs. These findings align with the burst term analysis from the Web of Science Core Collection, further supporting the view that rigorous, modern clinical trial designs are becoming a cornerstone of research in CHM for chronic pain. This transition represents a necessary step for the field to engage with international standards.

However, current clinical trial designs in this area still face notable limitations. A review of the literature reveals that most CHM clinical studies remain single-center and small in scale. Methodologically, they often lack the rigorous design and detailed reporting found in studies of comparable Western medical management. To meet international standards of evidence-based medicine, future research must consider adopting methodological frameworks from modern medicine, including larger sample sizes, multi-center designs, and objective, multifaceted outcome assessments. For example, the inclusion of validated pain rating scales is recommended. Additionally, due to the complex composition of many CHM formulations, long-term safety and follow-up must be emphasized. Real-world studies, as opposed to strictly controlled RCTs, may offer greater practical relevance for CHM due to its broad application and individualized nature. Consequently, complementing RCTs with large-scale, multi-center real-world research is likely to become an important trend.

#### 3.9.2 Chronic pain spectrum represented by osteoarthritis and migraine

The keywords “osteoarthritis” and “migraine” reinforce findings from reference burst analysis based on Web of Science Core Collection data. These two conditions represent primary foci for the clinical application of CHM in chronic pain management. Both diseases are prevalent and impose significant economic burdens, making them high-priority conditions in primary care settings (43, 44). This underscores the significant potential of CHM in managing chronic pain within primary care.

Interestingly, “cancer pain”—a concept with longstanding relevance in CHM—appears in a more peripheral position in both the PubMed clinical trial data and the co-occurrence analysis from the Web of Science database. However, in the analysis of highly cited literature (26), cancer pain-related articles have received notable academic attention. This suggests that while CHM research on cancer pain is robust at the basic science level, its clinical translation remains underdeveloped. The historical use of CHM in managing cancer pain, coupled with its dual focus on symptom relief and root-cause management, suggests great clinical potential (45–47). Accelerating the transition from basic research to clinical application in this area is expected to become a major focus of future research.

#### 3.9.3 Integrating traditional and modern medicine in chronic pain management

The keyword co-occurrence map reveals strong connections between “flunarizine,” “randomized controlled trial,” and “Zhengtian Capsule.” This indicates ongoing efforts to integrate traditional Chinese and modern medical approaches, to identify synergistic management strategies that offer patients more effective and personalized options (48).

Similarly, in clinical research on chronic pain, the combination of CHM and modern pharmaceuticals is being explored to provide more comprehensive care. For example, studies have shown that Nimodipine combined with Yufeng Ningxin tablets results in a significantly higher cure rate for migraine (49).

These findings support the hypothesis that future clinical research on CHM for chronic pain will increasingly focus on integrated therapeutic models, blending traditional and modern management. Areas of potential include CHM-assisted reduction in analgesic dosage, mitigating drug dependence and tolerance, lowering adverse effects, and improving overall management outcomes and quality of life.

## 4 Discussion

### 4.1 Research hotspots and frontiers

By examining keyword bursts, citation bursts, key CHM, and the progress of clinical trials, we can identify and anticipate emerging trends in the study of CHM for chronic pain management.

#### 4.1.1 Key CHM and its relevant chemical components

An analysis of key CHM reveals that *Tripterygium wilfordii Hook.f*, *Sophora flavescens Aiton*, *Conioselinum anthriscoides “Chuanxiong*,” and *Paeonia lactiflora Pall* are currently among the most prominent and widely studied herbs in this domain.

*Tripterygium wilfordii Hook.f* is widely used for chronic pain associated with immune-inflammatory conditions due to its potent immunosuppressive and anti-inflammatory properties (50–53). Studies have shown that four active monomeric compounds isolated from *Tripterygium wilfordii Hook.f* (triptolide, celastrol, demethylzeylasteral, and wilforgine) exhibit analgesic effects in experimental models. One of the key analgesic targets is believed to be the Nav1.7 sodium channel protein. Among these compounds, triptolide demonstrates significant efficacy in relieving both neuropathic and inflammatory pain, even at relatively low doses (53). In addition to its analgesic properties, triptolide has demonstrated marked anti-inflammatory, immunosuppressive, and anticancer activities (54). Therefore, it may offer effective therapeutic potential for chronic pain commonly associated with clinical conditions such as rheumatoid arthritis and cancer (55, 56). These findings suggest that triptolide and related compounds warrant consideration in drug screening and future research on chronic pain management.

*Sophora flavescens Aiton* has demonstrated the ability to alleviate both inflammatory and peripheral neuropathic pain (57). Mechanistic studies suggest that oxymatrine, a key bioactive compound in *Sophora flavescens Aiton*, inhibits oxidative stress and inflammation by suppressing the phosphorylation of p38 and Nuclear Factor Kappa-B p65 pathway proteins in mice (58). Additionally, *Sophora flavescens Aiton* (commonly known as Kushen) has a long-standing history of both internal and topical use in TCM. The external application of *Sophora flavescens Aiton* has already been documented in classical texts, such as the Treatise on Cold Damage and Miscellaneous Diseases (Shang Han Za Bing Lun), and remains a common practice in contemporary clinical settings (59, 60). The analgesic effect of topical Kushen for persistent pain may be attributed to oxymatrine’s ability to regulate inflammation, chemokine activity, and immune mediators, as well as to inhibit mRNA expression of Transient Receptor Potential Ankyrin 1 and Transient Receptor Potential Vanilloid ion channels (61). In cancer pain management, a Kushen-based Chinese medicine formulation, Fufang Kushen Injection, has been shown not only to relieve cancer-associated pain but also to inhibit tumor growth directly (62–64). The primary mechanism involves inhibition of Transient Receptor Potential Vanilloid-mediated, capsaicin-induced Extracellular Signal-Regulated Kinase phosphorylation and a reduction in tumor-induced proinflammatory cytokine production (62). It is important to note that cancer-related chronic pain can arise from nearly all types of cancer therapies, including surgery, radiation, hormone therapy, and chemotherapy (65). In recent years, the use of neoadjuvant therapies (such as preoperative chemotherapy or radiotherapy) has increased, which has led to a higher incidence of treatment-related chronic pain (65, 66). Much of this pain is localized to soft tissues, muscles, or joints (65). Notably, topical administration has been demonstrated to alleviate cancer-related chronic pain (67) effectively. Given the dual modality of *Sophora flavescens Aiton* (as both an oral and topical agent) and its potential to both relieve cancer pain and exert antitumor effects, it may serve as a promising adjunctive therapy. The external application of *Sophora flavescens Aiton*-based formulations or extracts to manage cancer-related chronic pain (including pain directly caused by cancer and that resulting from chemotherapy or radiotherapy) represents a potentially valuable frontier for future research. Currently, however, this area remains underexplored.

*Conioselinum anthriscoides “Chuanxiong”* is regarded in TCM as a key herb for treating headaches and is well known for its blood-activating and stasis-resolving properties (68). Clinically, it has been used for a long time in the treatment of various headache disorders (69). The significant anti-migraine effects of total alkaloids from *Conioselinum anthriscoides Chuanxiong* have been confirmed through their ability to increase levels of 5-hydroxytryptamine and its metabolite 5-hydroxyindoleacetic acid (70). Another major component, ligustrazine, has been shown to target dopamine D2 receptors, thereby reducing reactive oxygen species production and abnormal nociception in migraine rat models. These mechanisms suggest its potential in the effective management of chronic migraine (71).

*Paeonia lactiflora Pall* is believed to relieve inflammatory pain and regulate nervous system function through the anti-inflammatory and neuroprotective effects of paeoniflorin, thereby reducing nociceptive transmission and effectively relieving peripheral neuropathic pain and associated inflammatory pain in conditions such as diabetic foot (72). The analgesic mechanisms of paeoniflorin are thought to involve the activation of adenosine A1 receptors, as well as the potential modulation of several other pathways, including suppressors of cytokine signaling, the endogenous opioid receptor system, and anti-neuroinflammatory effects (73). However, current research into these latter three pathways remains in the exploratory stage, and further studies are required to establish their definitive roles in pain modulation and analgesia.

A shared pharmacological feature of these herbs is their anti-inflammatory activity, which underlies their analgesic effects. This underscores the anti-inflammatory mechanism of CHM as a central research focus and an emerging trend in the management of chronic pain. As such, these herbs have become focal points in current research and provide valuable reference points for developing clinically relevant therapies in chronic pain management.

#### 4.1.2 Spectrum of hot diseases and related mechanisms of CHM regulation

Recent reference bursts, the progress of clinical trials, authors with high publication counts, and keyword co-occurrence analyses indicate a growing scholarly interest in the application of CHM for managing chronic pain associated with arthritis. This trend suggests that research in this area will likely continue to expand in the coming years. Among the most prominent diseases under investigation are inflammatory arthritic conditions, particularly rheumatoid arthritis and osteoarthritis. As rheumatoid arthritis and osteoarthritis are the most common, both of which are the most prevalent types of arthritis (74, 75). In TCM, these conditions correspond to the category of “bone bi syndrome” (76, 77).

Osteoarthritis is a chronic degenerative joint disease primarily affecting older adults. It often results in joint damage and chronic pain and can lead to disability in severe cases (78). The associated chronic pain significantly impairs patients’ ability to perform daily activities and is considered one of the leading causes of chronic pain (79). Chronic pain caused by osteoarthritis has drawn increasing attention from researchers in recent years (80, 81). Particularly as rising obesity rates and population aging contribute to an increased prevalence of the condition (82). Currently, modern Western medicine lacks highly effective and specific therapeutic interventions for osteoarthritis (83). In contrast, CHM has demonstrated evidence-based efficacy in managing osteoarthritis-related chronic pain (84). Specifically, active compounds in CHM have been shown to attenuate inflammation mediated by key cytokines, such as interleukin and tumor necrosis factor, both of which are critical in the pathogenesis of osteoarthritis-associated pain (13, 85). Indeed, pro-inflammatory mediators are widely recognized as primary drivers of pain in osteoarthritis (86, 87). A real-world clinical study on knee osteoarthritis demonstrated that integrative treatment combining CHM with Western medicine significantly outperformed conventional treatment alone in managing chronic pain (88). This finding highlights the potential of integrative approaches as a promising direction for future research. According to TCM theory, pain is often associated with stagnation or obstruction of qi and blood. Therefore, herbal formulations aimed at promoting blood circulation and resolving blood stasis have demonstrated significant efficacy in alleviating certain types of pain. These effects have been substantiated in clinical studies on knee osteoarthritis (89), offering valuable guidance for chronic pain management in clinical practice.

Rheumatoid arthritis is a chronic, systemic autoimmune disease that primarily affects the synovial joints, often resulting in functional limitations and chronic pain (31). The global prevalence of rheumatoid arthritis is estimated to be approximately 0.24% (90). For patients with rheumatoid arthritis, pain remains the symptom they most hope to alleviate (91). However, there is still a lack of safe, effective, and relatively low-burden pharmacological options for pain management in this population (31). CHM has demonstrated potential in managing chronic pain associated with rheumatoid arthritis, primarily through the modulation of inflammatory mediators and inhibition of oxidative stress responses (92, 93). Notably, CHM may play a key role by preventing the nuclear translocation of Nuclear Factor Kappa-B, a mechanism that has been identified as critical in recent studies (94, 95). Nuclear Factor Kappa-B emerged as a high-frequency keyword in our bibliometric analysis, highlighting its significance as a research hotspot in studies of chronic pain. On the other hand, central sensitization is also recognized as an important factor in rheumatoid arthritis -related pain (96). This implies that even when inflammation is well controlled, some patients with rheumatoid arthritis may continue to experience inadequate pain relief (97). Acupuncture has demonstrated substantial evidence for its efficacy and underlying mechanisms in modulating central sensitization and, consequently, in supporting pain management (98–101). By contrast, similar research on CHM in this context remains limited. Therefore, the regulation of Nuclear Factor Kappa-B by CHM is currently a focal point in the management of chronic pain in rheumatoid arthritis. Future research frontiers may include the investigation of central sensitization mechanisms in pain management and the synergistic effects of combined CHM and acupuncture therapies.

Moreover, the high co-citation ranking of top-tier journals like *Annals of the Rheumatic Diseases* and *The Lancet* is particularly noteworthy. It signifies that CHM research is actively aligning with mainstream medical standards, citing landmark articles on diseases such as rheumatoid arthritis and osteoarthritis to establish scientific rigor and clinical relevance. This trend underscores a critical shift toward conducting more rigorous, comparative clinical trials and integrating CHM into evidence-based frameworks, which is essential for its future inclusion in international clinical guidelines.

#### 4.1.3 Hotpot and frontier tools in research

Notably, the recent emergence of keywords such as network pharmacology and molecular docking indicates that contemporary research in this field is increasingly integrating modern computational and systems biology approaches. These methodologies represent a shift toward more precise, mechanism-based investigations and signal a growing emphasis on exploring the molecular underpinnings of CHM management for chronic pain.

Beyond the current use of network pharmacology and molecular docking, the broader role of Artificial Intelligence (AI) offers profound inspiration for the field. Although it is not clustered as a core keyword, as far as we conclude, the importance of AI or large language models merits attention. This trend is exemplified by the selection of the 2024 Nobel Prize in Chemistry, where AlphaFold, an AI-driven tool, was recognized as a groundbreaking advancement and a research frontier in the life sciences (102). At present, AI has been utilized in various aspects of CHM research, including drug design, clinical decision support, data analysis, and disease characteristic identification and prediction (103–107). For example, AI can revolutionize CHM research by mining vast quantities of traditional and modern data to generate novel hypotheses, enabling highly personalized treatments through precise “pattern differentiation,” and modernizing clinical trials by identifying optimal patient subgroups (108).

This deeper integration of AI signifies a paradigm shift, advancing CHM from a traditional practice to a data-driven, precision-focused medical science.

#### 4.1.4 Regional bias and international generalizability

An analysis of institutional publication volumes reveals that eight of the top 10 publishing institutions are located in China’s economically developed coastal regions. In contrast, institutions in the central and western regions are relatively underrepresented, both in publication output and in collaborative research networks. Coastal institutions exhibit a clear advantage in both dimensions, demonstrating a pronounced “top-heavy” pattern in scholarly productivity.

Moreover, the concentration of CHM research in coastal regions may reflect disparities in research infrastructure, policy support, funding availability, and healthcare resources. For instance, the economically developed eastern coastal areas have significant advantages over the less-developed western regions in terms of TCM education, talent subsidies, and the availability of related medical positions, resulting in a greater aggregation of TCM professionals in these eastern coastal areas (109). In the field of CHM for chronic pain, this manifests as most affiliated institutions being concentrated in the developed eastern coastal regions. Also, due to the region-specific nature of TCM practices, research findings from coastal regions may not be universally applicable, particularly for guiding CHM-based pain management strategies in inland or under-resourced areas (110). So, there is a need to provide more support in the field of CHM for chronic pain in the central and western regions.

Compared to acupuncture and massage, which are increasingly recognized in the global management of chronic pain, CHM remains controversial in many parts of the world. One key reason lies in its deep historical roots in specific regions and the inherent complexity of its formulations (111). Consequently, the findings are likely to be more generalizable to countries such as Japan and South Korea, which share a longstanding tradition of CHM usage (112–115).

In these regions, both clinical application and academic research on CHM, particularly compound formulas, are active and well-established. Research in these areas often focuses on evaluating clinical efficacy and integrating approaches that combine Chinese and Western medicine (116–119). In this sense, the identification of key CHMs and the emphasis on real-world clinical validation can offer valuable insights to researchers in these countries. This focus may represent a current research hotspot and frontier in the study of CHM for chronic pain.

In recent years, interest in complementary and alternative medicine related to CHM has gradually increased in Western countries. For example, in France and Germany, nearly 50% of adults have tried some form of complementary or alternative medical treatment within a year (120). However, for Western countries where CHM lacks historical or cultural grounding, generalizability may be more limited. In these regions, researchers and clinical doctors tend to approach CHM more cautiously. Due to cultural differences and the relative lack of relevant research, studies in Western countries often emphasize purified components, single-compound analyses, the investigation of CHM toxicity and side effects, and mechanisms of action, areas that are more closely aligned with mainstream biomedical paradigms (111, 120, 121). Therefore, comprehensive research and mechanistic studies on CHM will be crucial for achieving greater recognition in the West and are likely to become future hotspots and frontiers in CHM research in these countries.

### 4.2 Strength and limitation

The increasing attention to chronic pain and the growing recognition of TCM suggest that research on CHM for chronic pain management holds significant development potential. Consequently, this study provides valuable insights and serves as an important foundation for further exploration.

However, it is necessary to acknowledge certain limitations inherent in this study. First, to ensure the inclusion of high-quality literature, to reduce limitations in structured export formats, which can introduce errors during bibliometric analysis, to align with the characteristics of the visualization tools used, and to enhance the credibility, standardization, and reproducibility of our findings, this study restricted the analyzed articles to those published in English. This exclusion may have led to the omission of some relevant literature, introducing a degree of selection bias. Future research should address this gap by methodically reviewing Chinese-language publications to provide a more holistic perspective.

Second, article types such as letters and case reports were excluded from this study. While this ensured the analytical integrity of the included literature, it may have inadvertently narrowed the scope of analysis and limited the breadth of perspectives considered.

Lastly, we must acknowledge that this study was not registered in a relevant international database before analysis, which may lead to the repeated analysis of studies on the same topic, in potential duplication of research.

Furthermore, maintaining active communication with researchers and frontline clinicians in the field of CHM for chronic pain management is crucial. Such collaboration would facilitate a deeper understanding of the latest advancements and foster a more objective, comprehensive, and nuanced evaluation of this evolving field.

## 5 Conclusion

This study methodically summarizes the current status, research hotspots, and emerging trends in the application of CHM for chronic pain management. The findings highlight the growing academic interest and increasing international recognition of CHM in this field. Future basic research is expected to focus more on the analgesic mechanisms of CHM, particularly its anti-inflammatory effects. Clinically, arthritis is likely to remain the primary disease area of focus for CHM-based pain management. In terms of clinical research, key frontiers will include the integration of traditional Chinese and Western medicine for chronic pain management, the clinical translation of CHM in cancer pain treatment, and real-world studies. Additionally, molecular docking, network pharmacology, and AI are anticipated to become prominent research tools and new driving forces in this field. Regional bias highlights the disparity in the distribution of research resources between the eastern and western regions of China. International generalizability discusses the specific applicability of CHM in chronic pain management in different countries.

## Data Availability

The original contributions presented in the study are included in the article/Supplementary material, further inquiries can be directed to the corresponding author.
