# Supplementary material for: Chinese herbal medicine for chronic pain: a bibliometric analysis based on integrated databases (2011–2024)
Source: Front Med (Lausanne). 2025 Aug 8;12:1642093. doi: 10.3389/fmed.2025.1642093 (PMC12370650; doi:10.3389/fmed.2025.1642093)
Supplement: Supplementary file 1 [file Data_Sheet_1.pdf]

1 Supplementary Tables

Supplemental Table 1. Summary of Data Sources and Selection Criteria from the Web of Science Core Collection Database

Category		Specific Standard requirement
Research database		Web of Science core collection
Citation indexes		Science Citation Index Expanded
Searching period		January 2011 to July 2024
Developed by		Xiangyu Wang, Yongfang Li, Haojin Cheng
Language		"English"
Keywords	Keywords for searching	TS = (Traditional Chinese Medicine OR Chinese Herbal Medicine OR TCM OR "Chinese medicine") AND TS = ("chronic pain" OR "chronic headache" OR "chronic migraine" OR "chronic tension-type headache" OR "fibromyalgia" OR "neuropathic pain" OR "chronic back pain" OR "osteoarthritis" OR "rheumatoid arthritis" OR "cancer pain" OR "complex regional pain syndrome" OR "persistent pain" OR "recurrent pain")
	Keywords for subject categories	"Integrative Complementary Medicine", "Pharmacology Pharmacy", "Chemistry Medical", "Medicine Research Experimental", "Medicine General Internal", "Neurosciences", "Geriatrics Gerontology", "Surgery", "Peripheral Vascular Disease", and "Clinical Neurology"
Data extraction		Export with full records and cited references in plain text format
Sample size		1310

Supplemental Table 2. Summary of Data Sources and Selection Criteria from the PubMed Database

Category	Specific Standard requirement
Research database	PubMed
Citation indexes	Science Citation Index Expanded
Searching period	January 2011 to July 2024
Developed by	Xiangyu Wang, Yongfang Li, Haojin Cheng
Language	“English”
Keywords	(("Drugs, Chinese Herbal"[Mesh] OR "Chinese Drug*"[Title/Abstract] OR "Chinese Herbal Drug*"[Title/Abstract] OR "Chinese Plant Extract*"[Title/Abstract]) AND ("Chronic Pain"[Mesh] OR "Chronic Pain*"[Title/Abstract] OR "Chronic Primary Pain*"[Title/Abstract] OR "Chronic Secondary Pain*"[Title/Abstract] OR "Headache Disorders"[Mesh] OR "Headache Disorder*"[Title/Abstract] OR "Cluster Headache"[Mesh] OR "Cluster Headache"[Title/Abstract] OR "Tension-Type Headache"[Mesh] OR "Tension-Type Headache"[Title/Abstract] OR "Fibromyalgia"[Mesh] OR Fibromyalgia*[Title/Abstract] OR "Neuralgia"[Mesh] OR Neuralgia*[Title/Abstract] OR "Back Pain"[Mesh] OR "Back Pain*"[Title/Abstract] OR "Osteoarthritis"[Mesh] OR Osteoarthriti*[Title/Abstract] OR "Cancer Pain"[Mesh] OR "Cancer Pain*"[Title/Abstract] OR "Neoplasm-Related Pain*"[Title/Abstract] OR "Complex Regional Pain Syndromes"[Mesh] OR "Complex Regional Pain* Syndrome*"[Title/Abstract] OR "Pain, Intractable"[Mesh] OR "Pain*, Intractable"[Title/Abstract] OR "Facial Pain"[Mesh] OR "Facial Pain*"[Title/Abstract])) AND English[lang] AND ("2011/01/01"[PDAT] :

	"2024/07/01"[PDAT])
Sample size	538

Supplemental Table 3. The BIBLIO checklist for reporting the bibliometric reviews of the biomedical literature

Section/Topic	Item No.	Checklist item	Reported on page No.
**Title**			
Identification	1	Identify the report as a bibliometric review in the title.	1
Issues/topics	2	Indicate the key issues/topics under investigation and coverage of time period.	1
**Abstract**			
Structured summary	3	Structured summary including (as applicable): background, methods, results (key findings) and conclusions.	1，2
**Introduction/ Background**			
Justification/ Rationale/ Explanation	4	Present review of existing knowledge and epidemiological information.	2，3
Objectives	5	Statement of the objective (s) or question (s).	3
**Methods**			
Search engines (data sources)	6	Describe all information sources (such as electronic databases, contact with study authors, trial registers or other grey literature sources).	3，4
Search strategy	7	Keywords and systematization criteria (date of search, language, type of document) for the search.	4
Time period	8	The period that the review covers and the justification.	3
Eligibility criteria	9	Describe all inclusion and exclusion criteria; languages; study design, type of publication and time period.	4
Data refinement (data selection procedure)	10	Remove the irrelevant articles; inspection to eliminate duplicate and unrelated articles (after evaluation of the title, abstract and content).	4
Quality assessment (optional)	11	Assessment of papers by three authors and the use of assessing checklists.	4

Data synthesis	12	Describe the methods used for summarizing, handling, synthesis, tabulations or schematic displays. Describe how the data were analysed.	4，5
**Results**			
Descriptive findings (statistics)	13	- Provide details of the search and selection process in a flow diagram.<br>- Number of citations retrieved (number of publication, year of publication, type of documents, country of publication, articles with the highest impact, most impactful authors, most impactful articles, authors with the highest production, top journals, top institutions, …)	5-13
Schematic map and trend	14	Summarize and/or present the schematic maps and trends using an appropriate software to present citations, journals, authors, top journals, time trends, emerging literature, and any relevant indicators (as applicable) [1-5].	5-13
Tabulation and summarizing the findings	15	General recommendation: Studies under consideration could be summarized and organized by different subtitles and different scenarios. Regardless, results need to be presented in separate tables covering each subtitle. The followings are some options that could help to summarize the findings.<br><br>*Option 1:*<br>- Start the presentation with a historical view [when and who first published on the topic].<br>- Report on review papers. The result should be listed in a separate table. Also, specify the review type (scoping review, narrative review, systematic review, and meta-analysis).<br>- Summarize the findings according to the study designs and main study types.<br>*Option 2:*<br>- Start the presentation with a historical view [when and who first published on the topic].<br>- Report on review papers. The result should be listed in a separate table. Also, indicate the review type (scoping review, narrative review, systematic review, and meta-analysis) should be specified.<br>- Summarize the findings according to outcome measures or populations. For example, see [6].<br>*Option 3:*<br>- Start the presentation with a historical view [when and who first published on the topic].	5-13

		- Report on review papers. The result should be listed in a separate table. Also, specify the review type (scoping review, narrative review, systematic review, and meta-analysis).<br>- Summarize the findings according to concept [7].<br>*Option 4.*<br>- Start the presentation with a historical view [when and who first published on the topic].<br>- Report on review papers. The result should be listed in a separate table, and also specify the review type (scoping review, narrative review, systematic review, and meta-analysis).<br>- Summarize the findings according to different subtitles relevant to the main topic [8].	
Synthesis of findings	16	Synthesize the findings as much as possible, find the gap, and propose a model, hypothesis, etc. (if applicable).	5-13
**Discussion**			
Summary of evidence	17	Summarize the main findings. The findings should be presented in more "general" or "accessible" terms.	13-19
Interpretation	18	Include interpretation consistent with results. Explanations for observed outcomes, similarities, and differences reported would be essential.	13-19
Strengths and limitations	19	Discuss the strengths and limitations.	19
Conclusion(s)	20	Provide a general interpretation of the results with respect to the review questions and objectives, as well as potential implications.	19-20

1. McDougal L, Dehingia N, Cheung WW, Dixit A, Raj A. COVID-19 burden, author affiliation and women's well-being: A bibliometric analysis of COVID-19 related publications including focus on low-and middle-income countries. eClinicalMedicine 2022; 52: 101606.
2. Henstock L, Wong R, Tsuchiya A, Spencer A. Behavioral theories that have influenced the way health state preferences are elicited and interpreted: A bibliometric mapping analysis of the ttime trade-off method with VOSviewer visualization. Front Health Serv 2022; 2: 848087.
3. Bodea F, Bungau SG, Negru AP, Radu A, Tarce AG, Tit DM, et al. Exploring new therapeutic avenues for ophthalmic disorders: Glaucoma-related molecular docking evaluation and bibliometric analysis for improved management of ocular diseases. Bioengineering 2023; 10(8): 983.
4. Sang XZ, Wang CQ, Chen W, Rong H, Hou LJ. An exhaustive analysis of post-traumatic brain injury dementia using bibliometric methodologies. Front Neurol 2023; 14: 1165059.
5. Ramli MI, Hamzaid NA, Engkasan JP, Usman J. Respiratory muscle training: a bibliometric analysis of 60 years' multidisciplinary journey. Biomed Eng Online 2023; 22(1): 50.
6. Akosman I, Kumar N, Mortenson R, Lans A, De La Garza Ramos R, Eleswarapu A,et al. Racial differences in perioperative complications, readmissions, and mortalities after elective spine surgery in the United States: A systematic review using AI-assisted bibliometric analysis. Glob Spine J 2023: 21925682231186759.

7. Tavousi M, Mohammadi S, Sadighi J, Zarei F, Kermani RM, Rostami R, Montazeri A. Measuring health literacy: A systematic review and bibliometric analysis of instruments from 1993 to 2021. Plos One 2022; 17(7): e0271524.
8. Montazeri A. Health-related quality of life in breast cancer patients: A bibliographic review of the literature from 1974 to 2007. J Exp Clin Cancer Res 2008; 27: 32.

Rights and permissions: The original source of the checklist is: Montazeri A, Mohammadi S, M.Hesari P, Ghaemi M, Riazi H, Sheikhi-Mobarakeh Z. Preliminary guideline for reporting bibliometric reviews of the biomedical literature (BIBLIO): a minimum requirements. *Systematic Reviews* 2023; 12: 239. doi.org/10.1186/s13643-023-02410-2 The article is licensed under a Creative Commons Attribution 4.0 International License (http://creativecommons.org/licenses/by/4.0/). A changes was made to the original checklist to add in full references to the cite
